# Expression Profiles and Interaction of MicroRNA and Transcripts in Response to Bovine Leukemia Virus Exposure

**DOI:** 10.3389/fvets.2022.887560

**Published:** 2022-07-19

**Authors:** Hao Ma, John D. Lippolis, Eduardo Casas

**Affiliations:** Ruminant Disease and Immunology Research Unit, National Animal Disease Center, Agricultural Research Service, United States Department of Agriculture, Ames, IA, United States

**Keywords:** gene Ontology (GO), differentially expressed transcripts (DET), white blood cell (WBC), bovine leukemia virus, transcript, microRNA, messenger RNA

## Abstract

Bovine leukemia virus (BLV) infection in cattle is omnipresent, which causes significantly economical losses worldwide. The objective of this study was to determine microRNA (miRNA) and transcript profiles and to establish their relationship in response to exposure to the virus. Small noncoding and messenger RNA were extracted and sequenced from serum and white blood cells (WBCs) derived from seven BLV seropositive and seven seronegative cows. Transcriptomic profiles were generated by sequencing RNA libraries from WBC. Bta-miR-206 and bta-miR-133a-3p were differentially expressed in serum (*P* < 0.05). In WBC, bta-miR-335-3p, bta-miR-375, and bta-novel-miR76-3p were differentially expressed (*P* < 0.03). There were 64 differentially expressed transcripts (DETs). Gene ontology (GO) analysis of the DETs overexpressed in the seropositive group with GOs of response to stimulus and immune system process predicted that the DETs could potentially negatively regulate viral life cycle and viral entry or release from host cells. In addition, the DETs depleted in the seropositive group could play a role in the downregulation of antigen processing and presentation of endogenous peptide antigen *via* MHC class I. The differentially expressed miRNAs targeted 17 DETs, among which the expressions of bta-miR-133a-3p and bta-miR-335-3p were significantly negatively correlated with the expressions of ENSBTAT00000079143 and ENSBTAT00000066733, respectively. Under high prediction criteria, 90 targets of the differentially expressed miRNAs were all non-DETs. The most enriched biological process GO term of the targets was the RNA-dependent DNA biosynthetic process, which could be associated with virus replication. These results suggested that the differentially expressed miRNAs fine-tune most of the target genes in responding to BLV exposure. In addition, Bta-miR-206 interacted with BLV regulatory genes *rex* and *tax* by targeting their coding regions. A further study of the miRNAs and the genes may reveal the molecular mechanisms of BLV infection and uncover possible ways to prevent the infection.

## Introduction

Bovine leukemia virus (BLV) is a retrovirus, naturally hosted in cattle and water buffalo (1, 2). The virus is able to infect several other animal species including sheep, goats, pigs, chickens, alpaca, rabbits, and rats (2–7). BLV infection in cattle is widespread worldwide (4, 6, 8). Most of the infected animals are asymptomatic; only <5% of BLV-infected cattle develop lymphosarcoma after 4–8 years of latency. There are three stages of the viral infection, namely, asymptomatic or aleukemic (serologically positive and persistent lymphocytosis negative), persistent lymphocytosis (serologically positive and persistent lymphocytosis positive), and leukemia or lymphoma formation (9, 10).

Bovine leukemia virus infection leads to significant economic losses by causing reduced milk production, premature culling, lymphosarcoma-related deaths, trade restrictions, and decreased resistance to infectious diseases in cattle (8, 10–12). Preventive and therapeutic strategies were developed against BLV infection including hematological, genomic, or serological methods to identify BLV-positive animals (13). Cattle with BLV are culled as well as corrective management and veterinary practices are indicated (13, 14). There is currently no effective vaccine against BLV (12, 13).

Attempting to find a solution to decrease the prevalence of BLV, an early study showed a considerable genetic influence on susceptibility to BLV infection (15). Recent studies on BLV-resistant cattle identified that DRB3 alleles correlate with a low proviral load profile (16–18). Based on the analysis of a US Holstein cattle population, the heritability estimate of leukosis incidence was approximately 8%, indicating that some genes may modulate leukosis (19). Furthermore, nine single nucleotide polymorphisms (SNPs) and the genes where these SNPs reside or neighbor have been associated with the incidence of BLV infection status (20). To further gain insight into the underlying mechanisms of the maintenance of latency, lymphoproliferation in the persistent lymphocytosis stage, and initiation of tumorogenesis, cattle cytokine imbalance has been analyzed during different stages of BLV infection. Results indicated that short-termed immune response of IL-12p40 and IFNγ followed by overexpression of IL-10 might modulate disease progression to persistent lymphocytosis (9, 21). In addition, epigenetic modulation of BLV expression revealed that methylation in the viral promoter may be associated with latency (22).

MicroRNAs (miRNAs) are an abundant class of small, conserved, noncoding RNA molecules that posttranscriptionally regulate gene expression *via* base-pairing with complementary sequences within messenger RNAs (23). Their importance in development, physiology, and disease in many organisms was identified (23–26). Our initial analysis reported that certain miRNAs were potentially associated with BLV infection (27). The objective of this study was to determine the association of miRNAs and expressed genes to establish putative candidate miRNAs that could potentially be used as biomarkers for the condition, based on the profiles of miRNA and transcript presented in BLV seropositive and seronegative samples.

## Materials and Methods

### Sample Test and Collection

Animal care and sample collection were performed according to the management protocol approved by the Institutional Animal Care and Use Committee of the National Animal Disease Center, in Ames, IA, United States.

The animals used in this study have been described previously (28). In brief, fourteen Holstein females were selected at the National Animal Disease Center, in Ames, IA, United States. Animals were randomly sampled from the dairy herd at the Center until seven positive and seven negative animals were identified. All animals included in this study were considered healthy according to the attending veterinarian. Samples came from 3 heifers and 11 cows with at least one calving and at midlactation. A jugular venipuncture with PAXgene tubes (PreAnalytiX GmbH, Hombrechtikon, Zurich, Switzerland) was used to obtain white blood cells from blood samples. Prior to RNA isolation, the tubes were incubated at room temperature for 2 h and placed in a refrigerator (4°C) before being centrifuged at 3,000 g for 10 min to collect serum and white blood cells (WBCs). Sera were obtained from a second blood sample collected through jugular venipuncture in serum separator vacutainer tubes (SST, TM BD, Franklin Lakes, NJ, United States). The tubes were incubated at 37°C for 30 min and then centrifuged at 1,250 g for 30 min. Isolated sera were stored at −80°C until processed. Sera were used to establish IgG reactivity to BLV with a direct enzyme-linked immunosorbent assay (ELISA), using the IDEXX Leukosis Serum X2 Ab Kit (Idexx Laboratories, Westbrook, ME, United States). A sample was considered positive if the sample-to-positive ratio (S/P) was >115%. The S/P was determined by the ratio of the difference between the optical density 450 nm (OD450) of the sample minus the OD450 of the negative control, divided by the difference between the OD450 of the mean positive control minus the OD450 of the negative control. Based on the ELISA test, seven animals were identified as seronegative, and seven were seropositive.

### RNA Isolation and Sequencing for Small RNA Analysis

The total RNA was extracted from serum, and WBC samples using the MagMAX^TM^ mirVana^TM^ Total RNA was eluted in 100 μl of RNase-free water. The concentration and quality of small RNAs in each sample were determined using a 10–40 nucleotide gate on an Agilent 2100 Bioanalyzer Small RNA chip (Agilent Technologies, Santa Clara, CA, United States).

The purified RNAs extracted from each sample were used to prepare individual libraries using the NEBNext Multiplex Small RNA Library Prep Kit (New England BioLabs, Ipswich, MA, United States). Library concentration and purification were performed using the QIAquick PCR purification kit (QIAGEN, Germantown, MD, United States). Each library was run on an Agilent 2100 Bioanalyzer High Sensitivity DNA chip (Agilent Technologies, Santa Clara, CA, United States) to determine the quality and quantity of the prepared library between 135 and 170 base pairs. Then, 30 ng of each library was pooled (14 libraries in the pool), and the size was selected using AMPure XP beads (Beckman Coulter, Indianapolis, IN, United States). Following the size selection, library pools were concentrated using the QIAquickPCR purification kit (QIAGEN, Germantown, MD, United States) and eluted in RNase-free water. The Agilent 2100 Bioanalyzer High Sensitivity DNA chip was used to determine the concentration of each library pool. The library pool was sequenced as single-end 50 base pair reads using the Illumina HiSeq 3000 System (Illumina, San Diego, CA, United States).

### RNA Isolation and Sequencing for Transcriptome Analysis

The WBC RNA was extracted using the mirVana^TM^ kit (Ambion, Carlsbad, CA). The quality of the mRNA was first checked using a Nanodrop (Thermo Fisher Scientific, Willington, DE). If samples had a measured λ260/λ280 > 1.9, they were run on Agilent 2000 bioanalyzer using the RNA 6000 Nano chip (Agilent, Santa Clara, CA). Samples with RIN # > 7.0 with 1–10 μg of total RNA were used to prepare libraries.

The libraries were constructed using the NEB Next Ultra RNA library kit for Illumina with the NEBNext PolyA Magnetic Isolation Module (NEB, Ipswich, MA). After final clean-up, 1 μl of the libraries were run on an Agilent 2000 bioanalyzer using the high sensitivity DNA chip. Based on the average size and concentration, the individual libraries were pooled to an equal molar concentration. The pools were sequenced using an Illumina HiSeq 3000 sequencer and were run at 2× 100 bp at the Iowa State University DNA Sequencing Facility (Ames, IA, USA).

### miRNA Analysis

The bovine reference genome was downloaded from Ensembl Genes 97 (https://uswest.ensembl.org/info/data/ftp/index.html), and the miRNA precursor and mature sequences were downloaded from miRBase (http://www.mirbase.org, Release 22.1). FastQC was used to evaluate serum and WBC small noncoding RNA raw sequences (29), and then cutadapt was used to select high-quality sequences (30). The serum and WBC small noncoding RNA sequences were counted using miRDeep2 version 2.0.0.8 (31). The counted BLV miRNA raw reads were then used to identify differentially expressed miRNAs by running the DESeq2 package (32). The small RNA sequences are available on the NCBI SRA under BioProject accession number PRJNA378560.

### Differentially Expressed Transcript

The WBC RNA sequencing reads were selected and evaluated in a similar way as small noncoding RNA, as previously described. Reads were mapped by STAR (33) against the bovine reference genome. Raw transcript sequences were sorted with samtools (34) and then counted with RSEM (35). Raw counts were analyzed using the DESeq2 package (32) to identify differentially expressed transcripts (DETs). Raw RNA-seq data were deposited in NCBI SRA under BioProject accession number PRJNA839936.

### Target Gene Prediction

The bovine 5′ and 3′ UTR and coding sequences were obtained by running the R package “biomartr” (Version 0.9.0) (36). The BLV noncoding 5′ and 3′ UTRs and coding sequences were extracted from the BLV genome deposited in NCBI (https://www.ncbi.nlm.nih.gov/assembly/GCF_000853665.1). Target genes of the differentially expressed miRNAs were separately predicted by miRanda and PITA (37, 38). The criteria to select target transcripts are maximum energy ≤-15 kcal/mol for miRanda and energetic score ≤-10 kcal/mol for PITA. The reported target genes were those that were predicted by both programs.

### Statistical and Gene Functional Analyses

Gene set enrichment was tested using the Fisher's exact test. Gene ontology (GO) of interested transcript sets was analyzed using OmicsBox (www.biobam.com). Spearman correlation parameters between the differentially expressed miRNA and transcript across the 14 animals were estimated using R (3.6.1).

## Results

### MicroRNA and Transcript Sequencing and Mapping

Small noncoding RNA sequencing from serum and WBC generated 568,377,432 clean reads (Table 1). Among these, 143,755,444 small RNA sequences from serum were mapped to the bovine genome (ARS-UCD1.2), and 2,758,717 were mapped to mature bovine miRNA. From WBC, 362,041,990 sequences were mapped to the bovine genome, and 140,595,431 were mapped to mature bovine miRNA. Therefore, a higher number of small noncoding RNA sequences were derived from WBC than from serum. WBC transcriptome sequencing produced a total of 759,903,436 sequences, of which 598,380,902 were uniquely mapped to the bovine genome and 106,416,066 sequences were mapped to multiple bovine loci.

**Table 1:** Sequencing and mapping statistics of small RNA and mRNA libraries.

**Sample**	**Serological**	**Serum small RNA sequencing**			**WBC small RNA sequencing**			**WBC mRNA sequencing**		
	**test**	**and mapping**			**and mapping**			**and mapping**		
		**Total reads**	**Mapped to bovine genome**	**Mapped to bovine miRNA**	**Total reads**	**Mapped to bovine genome**	**Mapped to bovine miRNA**	**Total reads**	**Uniquely mapped reads**	**Mapped to multiple loci**
1063	Positive	16,059,921	11,528,258	296,067	30,948,534	30,332,483	9,143,757	14,863,129	9,103,454	2,538,724
1085	Positive	11,321,246	7,705,964	141,594	29,846,729	29,173,912	12,361,518	49,104,702	29,203,024	8,750,363
1142	Positive	14,040,412	9,368,045	269,703	32,837,424	32,162,508	14,685,106	5,805,323	3,700,273	1,028,449
1146	Positive	17,673,286	13,308,511	201,134	21,547,420	20,997,325	5,390,817	44,111,661	34,361,904	5,658,942
1147	Positive	9,950,207	7,539,611	162,290	21,763,385	21,215,715	9,259,960	5,048,670	3,161,796	864,696
1304	Positive	11,816,051	8,764,456	189,169	34,925,647	34,249,823	9,126,233	34,983,778	28,985,487	4,656,321
1501	Positive	13,278,648	10,089,296	147,862	26,590,516	25,959,994	10,760,922	47,290,210	27,545,501	8,467,154
Seropositive total		94,139,771	68,304,141	1,407,819	198,459,655	194,091,760	70,728,313	201,207,473	136,061,439	31,964,649
1141	Negative	13,145,743	9,051,726	210,882	26,459,468	25,787,608	13,125,506	125,442,150	99,999,177	19,377,708
1143	Negative	14,192,603	10,771,104	149,012	26,701,404	26,135,954	10,980,643	92,134,530	78,760,568	10,488,536
1184	Negative	18,805,431	14,104,565	204,201	22,911,594	22,220,077	10,268,466	52,513,853	44,614,492	6,196,069
1202	Negative	12,915,970	9,988,116	229,307	28,592,582	27,688,500	13,133,045	63,885,214	55,595,414	7,371,294
1205	Negative	14,954,291	11,229,354	236,745	41,090,403	40,014,773	12,820,004	71,317,903	59,172,514	10,365,010
1306	Negative	14,451,563	10,023,778	142,791	16,544,142	16,186,089	5,836,390	76,572,024	65,225,167	8,703,528
1503	Negative	14,821,674	10,282,660	177,960	10,191,138	9,917,229	3,703,064	76,830,289	58,952,131	11,949,272
Seronegative total		103,287,275	75,451,303	1,350,898	172,490,731	167,950,230	69,867,118	558,695,963	462,319,463	74,451,417
Total		197,427,046	143,755,444	2,758,717	370,950,386	362,041,990	140,595,431	759,903,436	598,380,902	106,416,066

### Differentially Expressed miRNAs

Small noncoding RNA sequences were mapped to 836 and 1,086 mature miRNAs in miRBase with at least one read in serum and WBC, respectively (Supplementary Table S1). Novel miRNAs were predicted by miRDeep2 with at least 200 reads presented in at least 6 samples (39). A total of 22 and 252 novel miRNAs were identified in serum and WBC, respectively (Supplementary Table S2). Among those miRNAs, the mature miRNAs bta-miR-206, bta-miR-133a-3p, bta-miR-335-3p, and bta-miR-37 and one novel miRNA bta-novel-miR76-3p were differentially expressed (Table 2).

**Table 2:** Differentially expressed miRNAs in serum and white blood cell.

**Tissue**	**miRNA**	**Base mean**	**Log2 fold change**	***P*-value**	**FDR**
Serum	bta-miR-206	344	−4.0305	1.67E-05	1.85E-03
Serum	bta-miR-133a-3p	78	−3.3555	5.40E-04	4.97E-02
White blood cell	bta-miR-335-3p	94	−0.9050	2.42E-04	2.40E-02
White blood cell	bta-miR-375	2,115	2.3915	2.59E-04	2.40E-02
White blood cell	bta-novel-miR76-3p	48	8.9675	1.44E-13	5.32E-11

### Differentially Expressed Messenger RNA

**Figure 1:**
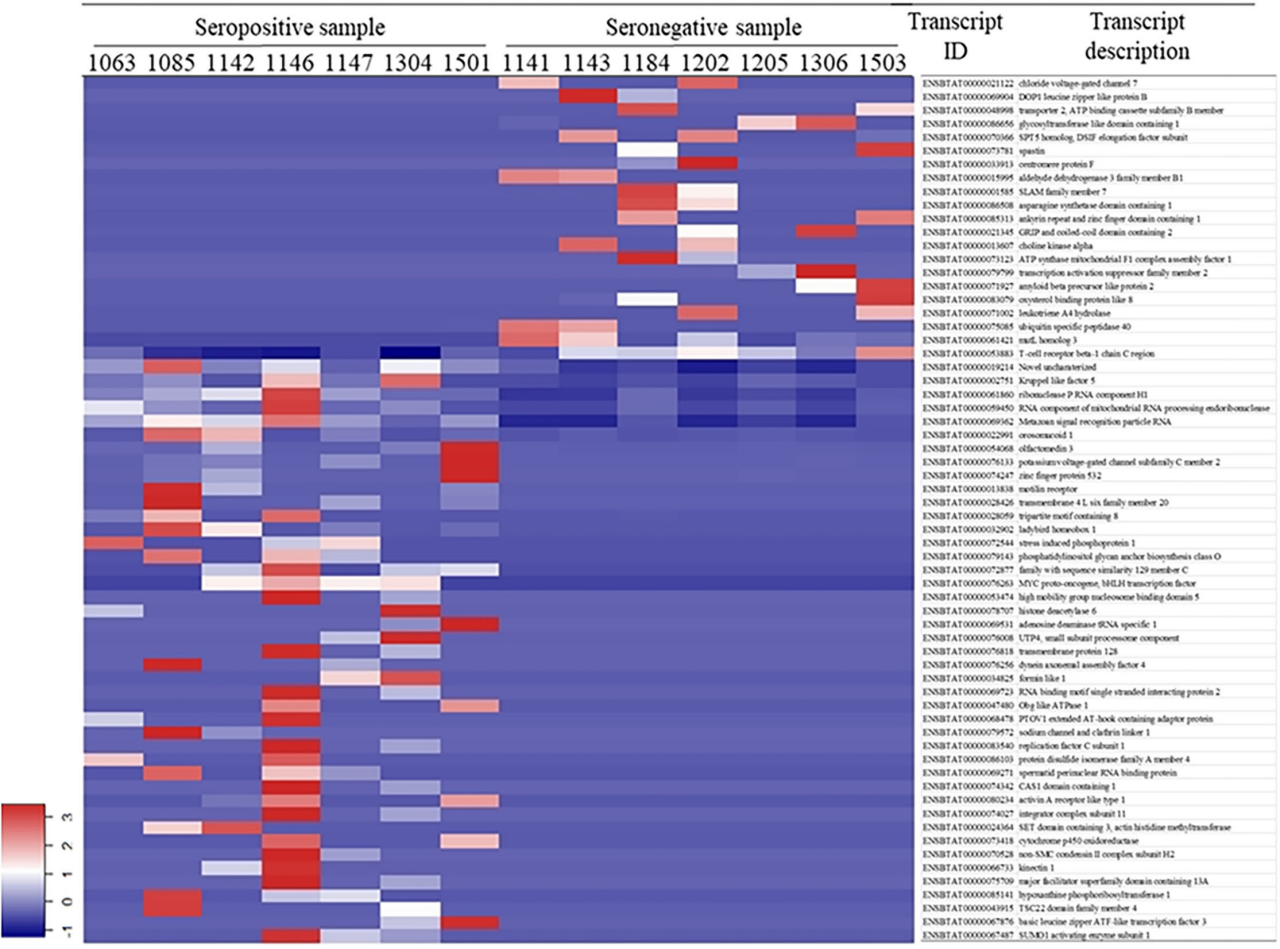
Differentially expressed transcripts in white blood cell.

White blood cell RNA sequences were aligned to 22,509 bovine transcripts with a raw sequence count >10 for the 14 samples, among which 64 transcripts were differentially expressed (Figure 1). There were 43 DETs that were upregulated in the seropositive group and 21 DETs upregulated in the seronegative group. A detailed description of the DETs is listed in Supplementary Table S3.

### *In silico* Functional Analysis of Differentially Expressed Messenger RNA

**Figure 2:**
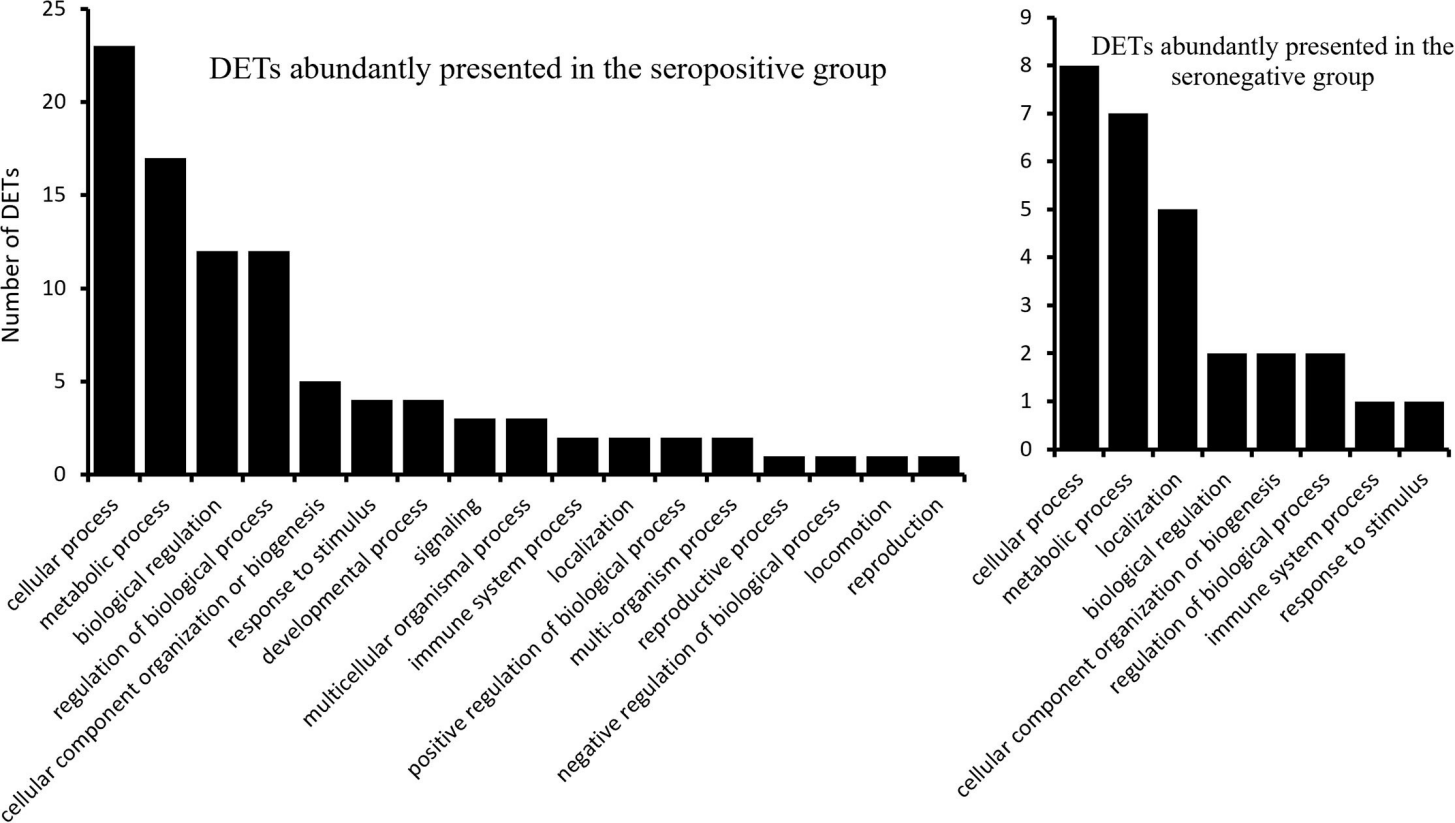
The biological processes (2nd level) of the differentially expressed transcripts.

Gene ontology analysis of the 43 DETs presented abundantly in the seropositive group revealed 17 associated biological processes at the second level of GOs (Figure 2). Transcripts ENSBTAT00000080234, ENSBTAT00000028059, ENSBTAT00000013838, and ENSBTAT00000083540 were associated with response to stimulus, and ENSBTAT00000022991 and ENSBTAT00000028059 were related to the immune system process. The specific biological processes of high-level GOs of those transcripts suggested that the animals might be actively responding to BLV infection through the regulation of gene expression. For example, the transcript ENSBTAT00000028059, encoded by a gene named *TRIM8*, is associated with both response to stimulus and immune system process. The specific biological processes of the transcript described by its high-level GOs include not only negative regulation of viral release and entry into the host cell, defense response, and innate immune response but also negative regulation of viral gene expression, viral process, viral transcription, and viral life cycle (Supplementary Table S4).

The 21 upregulated transcripts in the seronegative group share the same GO terms at the second level as the 43 transcripts from the seropositive group (Figure 2); however, the specificities of the GO terms are different from that of the 43 transcripts. For example, ENSBTAT00000048998, encoded by gene *TAP2* with the 2nd level GO of the immune system process, has high-level GOs without negative regulating viral-related biological processes, but involving antigen processing and presentation, transport, antigen processing and presentation of endogenous peptide antigen, antigen processing and presentation of peptide antigen *via* MHC class I, as well as organic substance, nitrogen compound, amide, and peptide transport (Supplementary Table S5).

### miRNA Target Analysis

With the mapped 22,509 cow transcripts as query, miRanda and PITA predicted 5,741 targets by the differentially expressed miRNAs (37, 38), seventeen of which were the DETs identified in this study (Supplementary Table S6). Among the 17 DETs, ENSBTAT00000075709 and ENSBTAT00000076256 were targeted at their 3′ untranslated regions (UTR); the remaining DETs were targeted in the coding region by the miRNAs. The coding regions of *GCC2-203, CENPF-201, KTN1-202*, and *OSBPL8-204* were separately targeted by two of the three differentially expressed miRNAs bta-miR-133a-3p, bta-miR-335-3p, and bta-miR-206 (Supplementary Table S6). Nine and eight targeted DETs were expressed abundantly in the seropositive and seronegative groups, respectively.

There are 5,724 of the target transcripts, which are non-DETs. When the criteria were increased to ≤-20 kcal/mol for miRanda and PITA, the number of non-DET targets decreased to 90 (Supplementary Table S7). The enrichment analysis of the 90 transcripts showed that all the significant GOs were overrepresented. RNA-dependent DNA biosynthetic process and RNA-directed DNA polymerase activity are the most significantly enriched GOs in the biological process and the molecular function categories, respectively (Table 3).

**Table 3:** The significantly enriched gene ontology terms of the 90 non-DET targets of the differentially expressed miRNAs.

**GO Category**	**GO ID**	**GO Name**	***P*-Value**	**FDR**
Biological process	GO:0006278	RNA-dependent DNA biosynthetic process	1.13E-27	8.99E-24
	GO:0071897	DNA biosynthetic process	3.92E-26	1.59E-22
	GO:0090305	Nucleic acid phosphodiester bond hydrolysis	1.35E-16	2.35E-13
	GO:0006259	DNA metabolic process	1.92E-14	2.59E-11
	GO:0044249	Cellular biosynthetic process	4.20E-09	4.25E-06
	GO:0009058	Biosynthetic process	9.18E-09	8.58E-06
	GO:1901576	Organic substance biosynthetic process	2.33E-08	1.89E-05
	GO:0034654	Nucleobase-containing compound biosynthetic process	2.21E-08	1.89E-05
	GO:0019438	Aromatic compound biosynthetic process	3.30E-08	2.51E-05
	GO:0034645	Cellular macromolecule biosynthetic process	3.60E-08	2.55E-05
	GO:0018130	Heterocycle biosynthetic process	3.78E-08	2.55E-05
	GO:0009059	Macromolecule biosynthetic process	4.49E-08	2.73E-05
	GO:1901362	Organic cyclic compound biosynthetic process	4.77E-08	2.76E-05
	GO:0044271	Cellular nitrogen compound biosynthetic process	1.13E-07	6.25E-05
	GO:0090304	Nucleic acid metabolic process	4.84E-07	2.56E-04
	GO:0006139	Nucleobase-containing compound metabolic process	2.72E-06	1.38E-03
	GO:0002396	MHC protein complex assembly	4.14E-06	1.57E-03
	GO:0002397	MHC class I protein complex assembly	4.14E-06	1.57E-03
	GO:0002583	Regulation of antigen processing and presentation of peptide antigen	4.14E-06	1.57E-03
	GO:0002584	Negative regulation of antigen processing and presentation of peptide antigen	4.14E-06	1.57E-03
	GO:0002590	Negative regulation of antigen processing and presentation of peptide antigen via MHC class I	4.14E-06	1.57E-03
	GO:0002589	Regulation of antigen processing and presentation of peptide antigen via MHC class I	4.14E-06	1.57E-03
	GO:0002501	Peptide antigen assembly with MHC protein complex	4.14E-06	1.57E-03
	GO:0002502	Peptide antigen assembly with MHC class I protein complex	4.14E-06	1.57E-03
	GO:0046483	Heterocycle metabolic process	4.45E-06	1.64E-03
	GO:0006725	Cellular aromatic compound metabolic process	4.64E-06	1.66E-03
	GO:1901360	Organic cyclic compound metabolic process	6.45E-06	2.24E-03
	GO:0034641	Cellular nitrogen compound metabolic process	9.65E-06	3.26E-03
	GO:0002577	Regulation of antigen processing and presentation	1.24E-05	3.87E-03
	GO:0002578	Negative regulation of antigen processing and presentation	1.24E-05	3.87E-03
	GO:0044238	Primary metabolic process	4.36E-05	1.29E-02
	GO:0044260	Cellular macromolecule metabolic process	4.54E-05	1.31E-02
	GO:0044237	Cellular metabolic process	5.02E-05	1.42E-02
	GO:0045721	Negative regulation of gluconeogenesis	8.64E-05	2.28E-02
	GO:0010677	Negative regulation of cellular carbohydrate metabolic process	1.15E-04	2.97E-02
	GO:0006807	Nitrogen compound metabolic process	1.29E-04	3.27E-02
	GO:0071704	Organic substance metabolic process	1.40E-04	3.47E-02
	GO:0008152	Metabolic process	1.44E-04	3.50E-02
	GO:0043170	Macromolecule metabolic process	1.59E-04	3.79E-02
Molecular function	GO:0003964	RNA-directed DNA polymerase activity	1.48E-27	8.99E-24
	GO:0034061	DNA polymerase activity	1.27E-25	3.87E-22
	GO:0016779	Nucleotidyltransferase activity	2.54E-21	6.16E-18
	GO:0140097	Catalytic activity, acting on DNA	2.04E-20	4.13E-17
	GO:0004519	Endonuclease activity	1.02E-15	1.54E-12
	GO:0004518	Nuclease activity	6.08E-13	7.39E-10
	GO:0016772	Transferase activity, transferring phosphorus-containing groups	4.01E-09	4.25E-06
	GO:0016740	Transferase activity	4.12E-08	2.63E-05
	GO:0023024	MHC class I protein complex binding	1.24E-05	3.87E-03
	GO:0016788	Hydrolase activity, acting on ester bonds	1.63E-05	4.95E-03
	GO:0023023	MHC protein complex binding	6.18E-05	1.71E-02
	GO:0008475	Procollagen-lysine 5-dioxygenase activity	8.64E-05	2.28E-02
	GO:0070815	Peptidyl-lysine 5-dioxygenase activity	1.84E-04	4.31E-02

The BLV genome consists of 8,714 nucleotides and encodes several structural and regulatory protein genes (40, 41). Both miRanda and PITA predicted that the BLV regulatory genes *rex* and *tax* were targeted by bta-miR-206 (Table 4).

**Table 4:** BLV genes targeted by bta-miR-206.

**miRNA**	**Target gene**	**Miranda**		**PITA**	
		**Max Score**	**Max Energy**	**Positions**	**Energetic score**
bta-miR-206	*rex*	140	−15.1	2,226	−12.99
bta-miR-206	*tax*	140	−15.1	2,179	−12.99

### Correlation Between Differentially Expressed miRNA and Their Targets

Spearman correlations were estimated between differentially expressed miRNAs and the target DETs. Among the 21 pairs of miRNA and transcript predicted by miRanda and PITA (Supplementary Table S6), the expressions of bta-miR-133a-3p and bta-miR-335-3p were significantly negatively correlated with the expressions of ENSBTAT00000079143 and ENSBTAT00000066733, respectively (*P* < 0.05) (Table 5), and the others presented trends without being significant. Both ENSBTAT00000079143 and ENSBTAT00000066733 were abundantly presented in the seropositive group.

**Table 5:** Spearman correlation coefficients between differencially expressed miRNA and transcript.

**Tanscript ID**	**Gene descriotion**	**Target region**	**bta-miR-206**	**bta-miR-133a-3p**	**bta-miR-335-3p**
ENSBTAT00000013607	Choline kinase alpha	Coding region	−0.1841		
ENSBTAT00000021345	GRIP and coiled-coil domain containing 2	Coding region		0.2816	0.3065
ENSBTAT00000028059	Tripartite motif containing 8	Coding region		−0.2039	
ENSBTAT00000033913	Centromere protein F	Coding region		0.0361	0.0180
ENSBTAT00000066733	Kinectin 1	Coding region	−0.4621		−0.5626^*^
ENSBTAT00000069271	Spermatid perinuclear RNA binding protein	Coding region			−0.5046
ENSBTAT00000070366	SPT5 homolog, DSIF elongation factor subunit	Coding region		−0.0398	
ENSBTAT00000071002	Leukotriene A4 hydrolase	Coding region		−0.0108	
ENSBTAT00000072544	Stress induced phosphoprotein 1	Coding region		−0.2419	
ENSBTAT00000072877	Niban apoptosis regulator 3	Coding region		−0.4093	
ENSBTAT00000074247	Zinc finger protein 532	Coding region		0.1206	
ENSBTAT00000075085	Ubiquitin specific peptidase 40	Coding region		0.3996	
ENSBTAT00000075709	Major facilitator superfamily domain containing 13A	3'UTR		−0.3881	
ENSBTAT00000076256	Dynein axonemal assembly factor 4	3'UTR	−0.2022		
ENSBTAT00000079143	Phosphatidylinositol glycan anchor biosynthesis class O	Coding region		−0.6980^**^	
ENSBTAT00000079799	Transcription activation suppressor family member 2	Coding region			0.4472
ENSBTAT00000083079	Oxysterol binding protein like 8	Coding region		0.1623	0.3762

* *p < 0.05;*

** *P < 0.01*.

## Discussion

The small RNA sequencing of the libraries identified two differentially expressed miRNAs in serum and three miRNAs in WBC. Those miRNAs are not overlapped, indicating that the cell-free circulating miRNAs in serum were not related to miRNAs in WBCs in the collected blood samples, but instead may be selectively released from different types of cells under varied pathological statuses (42). Among the five differentially expressed miRNAs, except for bta-novel-miR76-3p which have not been characterized, the other 4 miRNAs are all related to cancer.

The miRNA-206 expression has been reported to be significantly higher in human breast cancer tissue than that in paracancerous tissue (43). The human miRNA-206 inhibits both invasion and stemness of breast cancer and epithelial ovarian cancer (44, 45). In this study, the expression of bta-miR-206 was observed to be relatively higher in seronegative serum than in seropositive serum, which may have similar functions as in human. *In silico* prediction of this miRNA targets to BLV genome identified that it interacted with BLV regulatory gene *rex* and *tax* by targeting their coding regions, which provided a possible explanation of how this miRNA regulates BLV invasion. Similar to miRNA-206, miRNA-133a was also a tumor suppressor in breast cancer (46). Consistent with the human study, bta-miR-133a-3p shared a similar expression pattern as bta-miR-206 in this study. One of the target DETs of the miRNA, ENSBTAT00000028059, encoded by TRIM8 sowed negative regulation of viral life cycle, as well as viral entry and release from host cells by GO analysis. This result may deliver further insight into the molecular mechanisms of cancer-suppressing functions of bta-miR-133a. The differential expressions of miR-206 and miR-133a were also reported in swine which was related to the infection of Aujeszky's disease virus (47).

MicroRNA-335 was reported to be functionally the same as miRNA-206, which inhibits the migration of breast cancer through different mechanisms (43, 48). The expression of bta-miR-335-3p in WBC was significantly high in the seronegative animals and similar to bta-miR-206 and bta-miR-133a-3p in serum. miR-375 has been implicated in several types of cancers, including cervical cancer (49), skin cancer (50), and liver cancer (51). The miR-375 expression was high in Merkel Cell Carcinoma (MCC) cell lines and tissues compared with non-MCCs (50). A functional assay indicated that miR-375 can inhibit the proliferation and invasion of hepatocellular carcinoma cells (51). In this study, the expressions of bta-miR-375, as well as bta-novel-miR76-3p, were higher in seropositive WBC than that in seronegative WBC. In seropositive WBC, the expression of miRNA-375 was significantly high, while the expressions of miRNA-206 and miRNA-335 were significantly low, but these miRNAs were all reported to have cancer inhibition functions, which might indicate the dual functional roles of miRNAs (52–54).

From the normalized transcript counts, we observed a clear pattern between seropositive and seronegative groups (Figure 1). However, many transcripts showed that some individuals have similar reads in both groups. This may be due to specific characteristics of BLV infection; seronegative animals may also carry the virus but are at an early stage of infection. The seropositive animals might be at later phases of infection. Therefore, it is important to develop a method to accurately identify seropositive or seronegative animals at different infection stages.

Regulation of gene expressions in response to BLV infection was observed in our analysis. *In silico* functional analysis of the abundant DETs in the seropositive group showed that the expressions of some genes might encounter the viral infection through negative regulation of the viral life cycle and interaction with host cells (Supplementary Table S4). In contrast, the abundant DETs in the seronegative group suggested that some genes of the animals fight viral infection through antigen processing and presentation of endogenous peptide antigens (Supplementary Table S5); therefore, the animals probably initiated their different defense strategies under the different stages of viral infection by altering their immune-related gene expression (55).

MicroRNAs as key regulators in growth and development have been demonstrated in animals and human (56–59). In response to pathogen infection, miRNAs fine-tune various physiological and pathological processes (60–62). The complex roles of miRNAs are not only to suppress gene expression but also to activate gene expression in particular contexts (63–65). Our Spearman correlation analysis results in two significantly negative correlations among the expression values of 21 pairs of miRNA and target transcript, which implicated a big impact of some miRNAs on BLV infection as reported in other organisms (57, 58, 66). In contrast, the majority of the correlations between miRNAs and their targets were not significant, which might indicate miRNA's small effects on a large number of target genes (67). It is also possible that different BLV infection stages of the studied animals may alter the effects of the miRNAs. Furthermore, our results suggested that the miRNAs were not always significantly associated with their targets, and *in silico* predictive programs may not guarantee to identify significantly associated target genes.

The differentially expressed miRNAs targeted 3′ UTR, 5′ UTR, and coding regions of the cow transcripts in this study. Only 17 of the target genes were differentially expressed, but 5,724 targets were not differentially expressed. This result demonstrated the fine-tune characteristics of the miRNAs to their targets. Gene set enrichment analysis of the 90 target transcripts under high selection criteria for miRanda and PITA indicated that the significantly enriched GOs were all overrepresented (Table 3). The GOs of RNA-dependent DNA biosynthetic process and RNA-directed DNA polymerase activity are the most significantly enriched in the biological process and the molecular function categories, respectively (Table 3). These results may indicate the augmentation of the RNA virus in infected cattle. It has been reported that *Epstein Barr Virus* encoded EBNA1 could hinder the presentation of MHC I-associated peptides (68, 69). Similarly, our results suggested that BLV has employed some strategies to negatively regulate antigen processing and presentation of peptide antigen *via* MHC class I (Table 3); therefore, it might overcome the recognition of the infected cell and enable viral propagation in the host. The genes in the related processes were not significantly expressed but targeted by some of the differentially expressed miRNAs.

## Data Availability Statement

The datasets presented in this study can be found online repositories. The names of the repository/repositories and accession number(s) can be found below: https://www.ncbi.nlm.nih.gov/, PRJNA378560 and PRJNA839936.

## Ethics Statement

The animal study was reviewed and approved by Institutional Animal Care and Use Committee, National Animal Disease Center.

## Author Contributions

EC and JL conceived and designed the experiment. HM analyzed the data and wrote the manuscript. HM and EC interpreted the results. HM, JL, and EC reviewed and edited the manuscript. All authors contributed to the article and approved the submitted version.

## Funding

This research was part of an intramural project of the USDA/ARS National Animal Disease Center. The USDA had no role in study design, data collection, analysis, interpretation of results, or preparation of the manuscript.

## Conflict of Interest

The authors declare that the research was conducted in the absence of any commercial or financial relationships that could be construed as a potential conflict of interest.

## Publisher's Note

All claims expressed in this article are solely those of the authors and do not necessarily represent those of their affiliated organizations, or those of the publisher, the editors and the reviewers. Any product that may be evaluated in this article, or claim that may be made by its manufacturer, is not guaranteed or endorsed by the publisher.

## Abbreviations

BLV: bovine leukemia virus
miRNA: microRNA
GO: gene ontology
DET: differentially expressed transcripts
WBC: white blood cell
MCC: Merkel cell carcinoma
SNP: single nucleotide polymorphism
UTR: untranslated region.
